# Retraction Note: Auraptenol attenuates vincristine-induced mechanical hyperalgesia through serotonin 5-HT_1A_ receptors

**DOI:** 10.1038/s41598-019-39766-2

**Published:** 2019-03-18

**Authors:** Yunfei Wang, Shu-e Cao, Jianmin Tian, Guozhe Liu, Xiaoran Zhang, Pingfa Li

**Affiliations:** 1 Department of Anesthesiology, First Affiliated Hospital, Xinxiang Medical University, Weihui, Henan 453100 China; 2 Department of Medical Laboratory Science, Xinxiang Medical University, Weihui, Henan 453100 China

Retraction of: *Scientific Reports* 10.1038/srep03377, published online 29 November 2013

The editors are retracting this Article because the figures and the majority of the text are identical to those of another paper^1^. These issues undermine confidence in the validity of the study and the conclusions cannot be considered reliable.

The authors could not be reached.
